# Comparison of 3M Reston™ Self-Adhesive Foam Pad and 3M Microfoam™ Surgical Tape in Preventing Nasal Pressure Injuries Associated With Nasotracheal Intubation: A Randomized Controlled Trial

**DOI:** 10.7759/cureus.82045

**Published:** 2025-04-10

**Authors:** Mayumi Hashimoto, Naoko Tachi, Izumi Kuroda, Yoko Okumura, Yuji Kamimura, Kyoko Shida, Takuro Sanuki, Hidetaka Kuroda, Shota Tsukimoto, Hironori Miyamoto, Kenichiro Ishibashi, Hiroko Kobayashi, Yasuyuki Shibuya, Kazuya Sobue, Aiji Sato-Boku

**Affiliations:** 1 Department of Anesthesiology, Aichi Gakuin University, Nagoya, JPN; 2 Department of Anesthesiology and Intensive Care Medicine, Nagoya City University Graduate School of Medical Sciences, Nagoya, JPN; 3 Department of Dental Anesthesiology, Medical and Dental Sciences, Graduate School of Biomedical Sciences, Nagasaki University, Nagasaki, JPN; 4 Department of Dental Anesthesiology, Kanagawa Dental University, Kanagawa, JPN; 5 Department of Anesthesiology, Kanagawa Dental University, Kanagawa, JPN; 6 Department of Oral and Maxillofacial Surgery, Nagoya City University Graduate School of Medical Sciences, Nagoya, JPN; 7 Departmnet of Oral and Maxillofacial Surgery, Nagoya City University Graduate School of Medical Sciences, Nagoya, JPN

**Keywords:** medical device-related pressure injury, nasal pressure ulcer, nasotracheal intubation, pressure redistribution, randomized trial

## Abstract

Background: Nasotracheal intubation (NTI) is widely used in dental and maxillofacial surgeries to secure the airway while maintaining an unobstructed surgical field. However, NTI is associated with complications such as nasal bleeding, bacteremia, and retropharyngeal perforation. Among these, medical device-related pressure injuries (MDRPIs) caused by nasotracheal tubes are a significant concern. These injuries range from erythema to severe necrosis and can lead to postoperative pain, aesthetic concerns, and prolonged treatment. 3M Microfoam™ surgical tape (3MM; 3M Japan Limited, Shinagawa-ku, Tokyo) has been shown to effectively reduce the risk of nasal pressure injuries. However, its direct application to the skin can irritate, limiting its use in patients with sensitive skin. 3M Reston™ self-adhesive foam pads (3MR; 3M Japan Limited) are constructed using a breathable sponge designed to distribute pressure evenly. They are applied to the medical device rather than directly to the skin. This study aimed to compare the effectiveness of 3MR and 3MM in preventing nasal pressure injuries during NTI.

Methods: A randomized, double-blind prospective study was conducted with 144 patients who underwent general anesthesia with NTI for oral and maxillofacial surgery. Patients were randomly assigned to receive nasal alar protection with either 3MR or 3MM, both of which were attached to the endotracheal tube. The primary outcome was the incidence of nasal pressure injuries. These were classified as tube imprint, tube-induced erythema, and protective material-induced erythema. Those with no nasal pressure injuries constituted a fourth group classed as no imprint or erythema. The secondary outcomes were the pressure between the tube and the nasal alar (PTN) and the difference between the nostril diameter and the outer diameter of the intubation tube.

Results: Among the 140 patients analyzed, the proportion of patients with no imprint or erythema was higher in the 3MR (45 patients) than the 3MM (33 patients) group. The incidence of tube-induced erythema was lower in the 3MR group, but protective material-induced erythema was slightly higher in the 3MM group. However, the differences between the groups were not statistically significant (p = 0.66). The median PTN was 51 (30.1-75.9) g in the 3MR group and 58.1 (36.1-116.8) g in the 3MM group, which was not a significant difference (p = 0.08). The difference between nostril diameter and the outer diameter of the intubation tube was similar in both groups (p = 0.73).

Conclusion: Although no statistically significant differences between the 3MR and 3MM groups were observed in this study, the 3MR group showed a tendency to fewer pressure injuries and better pressure distribution. Given the importance of even pressure distribution to the prevention of MDRPIs, 3MR may be a moderately better alternative to 3MM for patients at high risk of nasal pressure injuries. Further studies with larger sample sizes and long-term follow-up are necessary to fully evaluate the clinical benefits of 3MR.

## Introduction

Comprehensive guidelines for pressure injury prevention have been established by the National Pressure Ulcer Advisory Panel, the European Pressure Ulcer Advisory Panel, and the Pan-Pacific Pressure Injury Alliance. In 2016, these organizations introduced significant revisions, replacing “pressure ulcer” with “pressure injury” and officially categorizing medical device-related pressure injuries (MDRPIs) and mucosal membrane pressure injuries as distinct types [1].

Among the medical devices that can cause these injuries, nasotracheal tubes have been identified as a major contributor to MDRPIs, and there is a need for effective protective measures when these are used [2-4]. Nasal pressure injuries are a common complication of nasotracheal intubation (NTI), occurring in 10%-50% of cases and ranging from erythema to severe necrosis [5-8]. These injuries can lead to postoperative pain and aesthetic issues, and can prolong treatment, significantly affecting patient recovery [5-8]. Given the frequency and impact of these injuries, effective preventive strategies are crucial.

A meta-analysis by Hoshijima et al. demonstrated that nasal protection strategies significantly minimize the occurrence of nasal pressure injuries during NTI. However, the small number of trials that have been conducted limits the validity of this finding, and a trial sequential analysis could not be performed in the meta-analysis. Therefore, more research is needed [9].

Among the existing preventive measures is 3M Microfoam™ surgical tape (3MM: 3M Japan Limited, Shinagawa-ku, Tokyo), which has been shown to effectively reduce the incidence of nasal pressure ulcers during NTI [10]. However, because it is applied directly to the skin, it can cause redness and irritation, making it unsuitable for patients with sensitive skin. 

This study aims to compare 3MM to the 3M Reston™ self-adhesive foam pad (3MR: 3M Japan Limited). This is an alternative to 3MM made of a breathable sponge material and designed to distribute pressure effectively. Unlike conventional pressure-relief materials, its adhesive surface is applied to the medical device rather than directly onto the skin, minimizing skin irritation. Given these properties, we hypothesize that 3MR could be an effective alternative for preventing nasal pressure injuries during NTI. If successful, this approach could contribute to safer, more comfortable NTI with a reduced risk of pressure injuries.

The primary objective of this study was to compare the effectiveness of 3MR and 3MM in preventing nasal pressure injuries during NTI. To assess the effectiveness, we focused on the following specific metrics: incidence of nasal pressure injuries (e.g., erythema, tube imprint), pressure distribution between the tube and the nasal alar, and skin irritation caused by the protective materials (3MR and 3MM).

## Materials and methods

**Figure 1:**
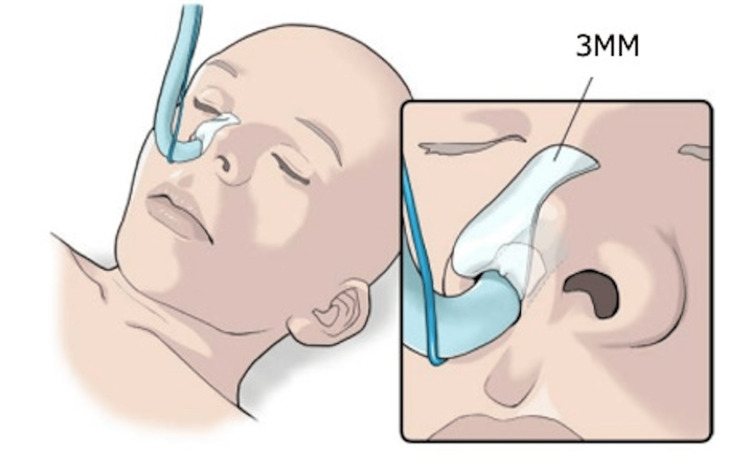
Protection method of the nasal wing in the previous study. Image credit: © MEDICAL FIG (https://medicalfig.medicaleducation.co.jp) In the previous study, 3M Microfoam™ surgical tape (3MM) was applied directly to the skin [10].

**Figure 2:**
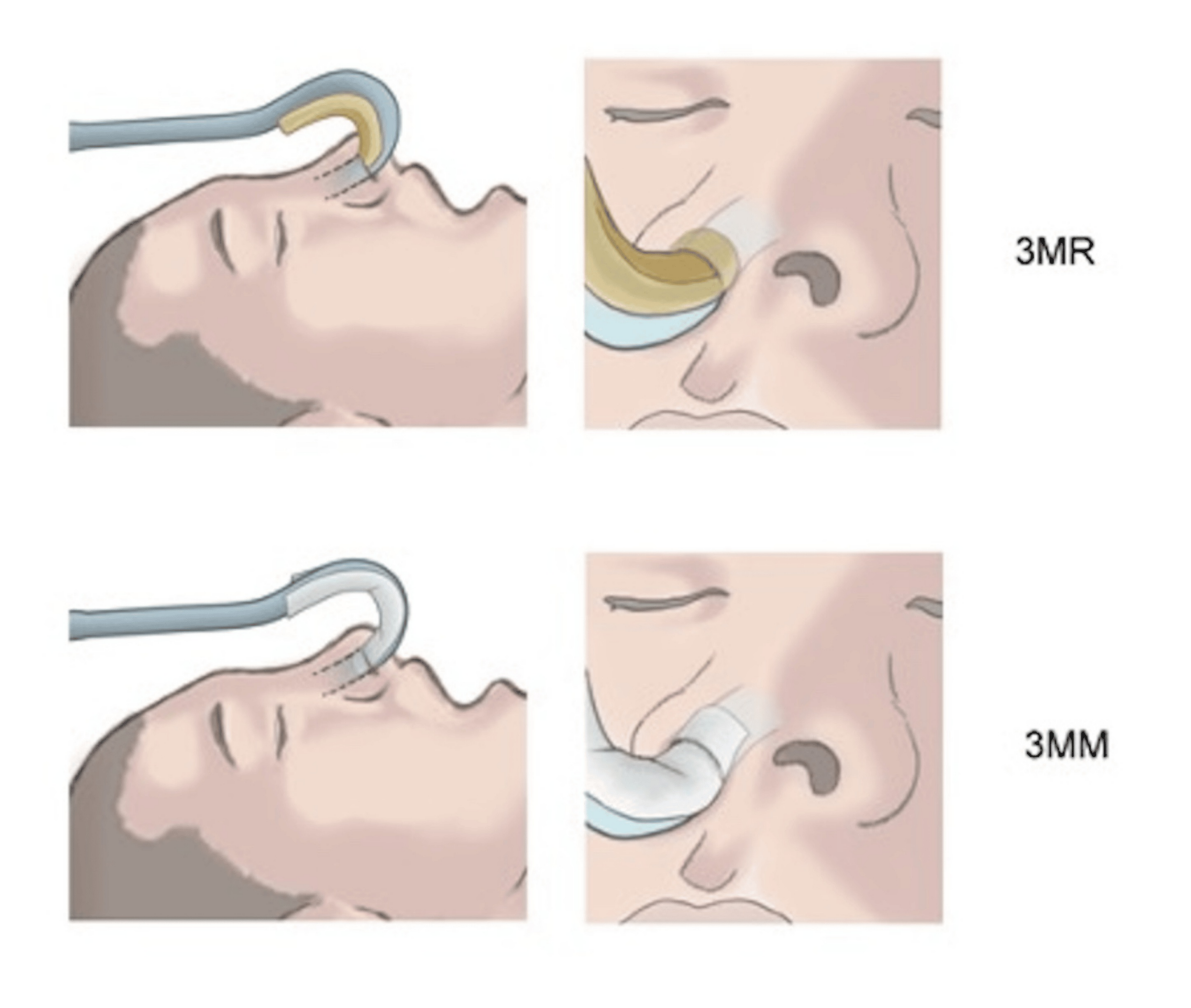
Protection method of the nasal wing in this study. Image credit: © MEDICAL FIG (https://medicalfig.medicaleducation.co.jp) Both 3MR and 3MM were directly attached to the endotracheal tube.

This prospective, randomized, double-blind study was approved by the Ethics Committee of the School of Dentistry, Aichi Gakuin University (approval no. 675) and was prospectively registered as a clinical trial with UMIN-CTR on December 20, 2023 (registration no UMIN000053153). It complies with the CONSORT guidelines and follows the principles outlined in the 2013 revision of the Declaration of Helsinki. The first patient was enrolled on January 4, 2024. Written informed consent to participation was obtained from all participants. The cohort consisted of 144 patients aged 20-70, classified as American Society of Anesthesiologists-physical status (ASA-PS) class 1 or 2, scheduled to undergo general anesthesia with NTI for oral and maxillofacial surgery. The exclusion criteria were individuals with sensitive skin and those with a history of nasal surgery. Patients who did not consent were also excluded. Both patients and evaluators were blinded to which group each patient was in. Participants were randomly allocated to receive nasal alar protection using either 3MR or 3MM, both of which were affixed to the endotracheal tube. Unlike previous studies, where protection was directly attached to the skin (Figure 1), in this study, both 3MR and 3MM were attached directly to the endotracheal tube (Figure 2). To protect the nasal alar during NTI, the 3MR or 3MM was intentionally inserted into the nasal cavity through the nostril. To minimize the risk of mucosal irritation, the material was positioned carefully to reduce the area of direct contact with the nasal mucosa.

Randomization was performed using a computer-generated sequence by a researcher uninvolved in the study to minimize bias and enhance reliability. A uniform anesthesia protocol was applied to all patients. Standard monitoring was performed throughout the procedures, including electrocardiography (ECG), and measures of blood pressure and oxygen saturation. Anesthesia induction was achieved using propofol (3 μg/mL target-controlled infusion), remifentanil (0.2 μg/kg/min), and fentanyl (100 μg), with rocuronium (0.6 mg/kg) as the neuromuscular blocking agent.

After induction, mask ventilation with 100% oxygen was maintained using propofol and remifentanil. The nasal mucosa and inferior nasal passages were thoroughly disinfected with benzalkonium (ZALKONIN solution 0.025, Kenei Pharmaceutical Co., Ltd, Osaka, Japan) [11], and tramazoline was applied to reduce bleeding during NTI [12].

To minimize nasal bleeding, NTI was performed through the right nostril [13]. All intubations were performed using a standardized technique by experienced anesthesiologists. The intubation process was generally uneventful, and there were no cases where intubation was considered difficult. The nasal alar was protected using either 3MR or 3MM. Applying 3MR and 3MM to the tracheal tube is a simple and straightforward procedure that does not require any special skills. Both materials can be easily attached by medical staff with basic training, making them feasible for routine clinical use.

The nasotracheal tube used in this study was the Polar™ Preformed Tracheal Tube (Smith Medical Japan Ltd, Tokyo, Japan), with an internal diameter of 7.0 mm for men and 6.5 mm for women. To ensure the stability of the tracheal tube during surgery, standard fixation techniques were used. The tube was secured with adhesive tape applied around the tube and fixed to the patient's cheek. This method minimized the risk of tube displacement and the occurrence of MDRPIs. Care was taken to avoid excessive tension on the tube to reduce pressure on the nasal alar.

The primary outcome was the occurrence or absence of nasal pressure injuries, assessed from the nasal tip to the nasal alar. Following extubation, an evaluation was conducted by an operating room nurse who was blinded to the patient’s group assignment (3MR or 3MM). After intubation, the nasal cavity and surrounding areas were carefully inspected to check for any injuries, and no immediate post-intubation injuries were observed. Patients were classified as having a tube imprint, tube-induced erythema, protective material-induced erythema, or no imprint or erythema. Tube-induced erythema and protective material-induced erythema were distinguished based on the location of the redness. Tube-induced erythema was identified when redness appeared on the nasal alar, where the nasotracheal tube itself was in contact. In contrast, protective material-induced erythema was classified when redness was observed on the nasal tip, where the 3MM or 3MR was in contact.

The secondary outcomes were the pressure between the tube and nose (PTN) and the difference between the nostril diameter and the outer diameter of the intubation tube. The difference between the nostril diameter and the outer diameter of the intubation tube was measured before attaching the protective material (3MR or 3MM). This measurement was conducted to ensure that there was sufficient space to accommodate the protective material. Therefore, the thickness of the protective material itself did not influence the measurement. Other variables included in our analysis were patients’ clinical and demographic characteristics, operative time, and anesthesia time.

**Figure 3:**
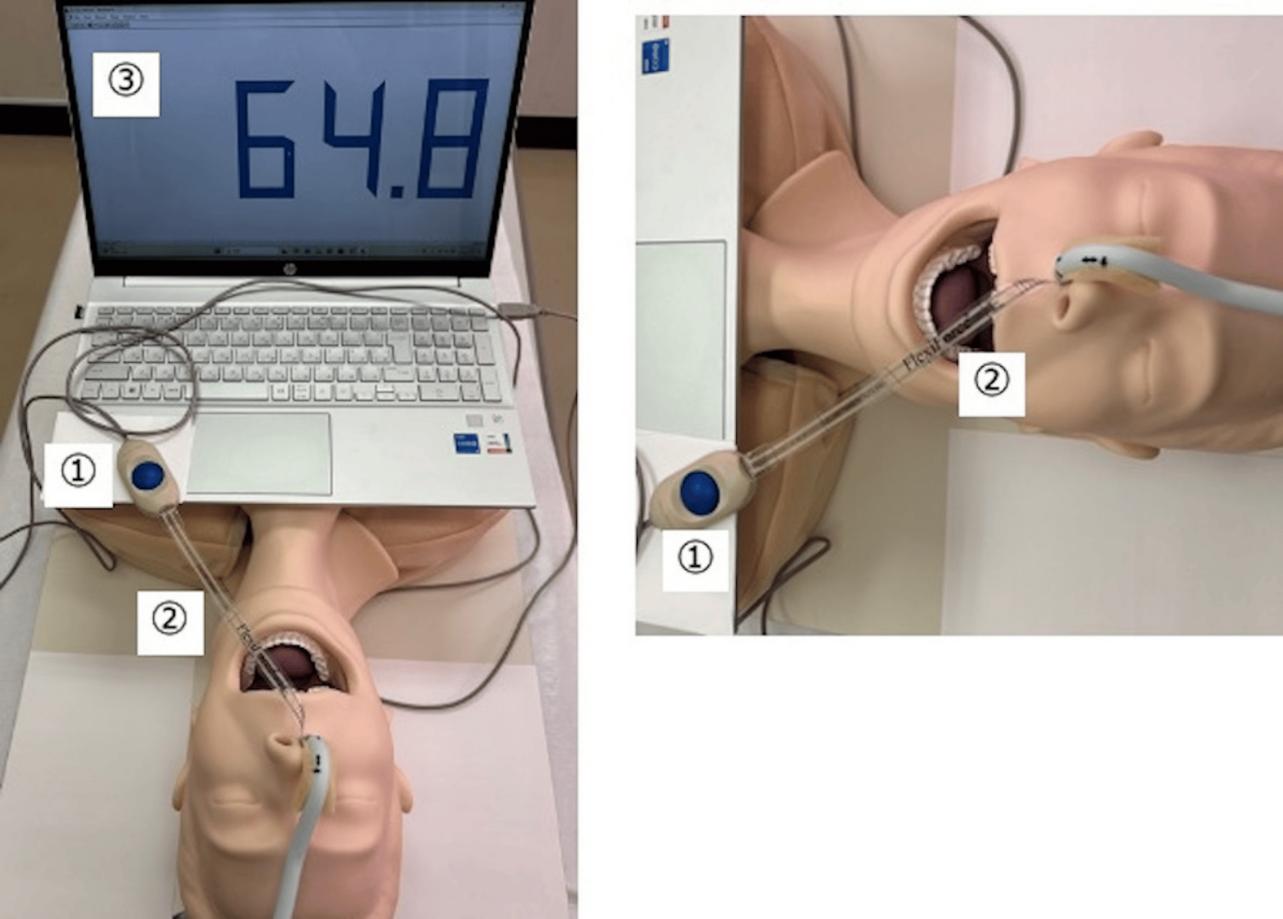
FlexiForce ELF. The FlexiForce ELF consists of a sensor handle (①), a sensor sheet (②), and software (③). The sensor sheet was inserted into the nasal cavity to measure PTN. The figure shows a mannequin for demonstration, but the actual measurements were conducted on patients. Image Credits: Aiji Sato.

The PTN was measured using a FlexiForce ELF (Tekscan Inc., Norwood, MA, USA). The FlexiForce ELF measurement was performed only once immediately after tracheal intubation to ensure consistency across all patients. Continuous measurement during surgery was not conducted, as changes in position and neck movement could have affected the pressure readings. The FlexiForce ELF consists of a sensor handle, a sensor sheet, and software (Figure 3). 

**Figure 4:**
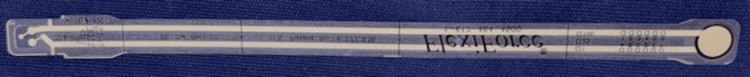
FlexiForce interface. FlexiForce interface pressure sensor is an ultrathin (0.203 mm), flexible, printed circuit. Its width and full length are 14 mm and 228.6 mm, respectively. At the end of the sensor is a circular probe (9.53 mm in diameter), which is defined by the silver circle on top of the pressure-sensitive ink. Image Credits: Aiji Sato.

The sensor used in this study was the FlexiForce interface pressure sensor (FlexiForce standard model B201, Tekscan Inc.) (Figure 4).

This is an ultrathin (0.203 mm) sensor with a flexible printed circuit. It is 14 × 228.6 mm and has a circular probe (9.53 mm in diameter) at the end, with a silver ring over the pressure-sensitive ink. Silver traces extend from the sensing area to the connector at the opposite end, forming conductive leads. Under controlled conditions, the FlexiForce sensors exhibit a low linearity error of ±3%, repeatability of ±2.5% of the full scale, drift of less than 5% per logarithmic time scale, and hysteresis below 4.5% of the full scale. Its response time is <5 μs, and it has a low temperature sensitivity of 0.36% per degree Celsius.

A power analysis with an effect size of 0.45, α error of 0.05%, and statistical power (1−β) of 80% found that a minimum of 130 patients was necessary. The effect size calculation was based on the results of a pilot study, using the distribution of nasal pressure injuries from the nasal tip to the nasal alar post-extubation as a reference (3MR, n = 10; 3MM, n = 10). The final sample size was adjusted for an expected dropout rate of 5%. The adjustment formula Nd = N/(1−R)² was applied, where N represents the original sample size and Nd accounts for the expected dropouts [14]. After these adjustments, 144 patients were recruited.

In our statistical analysis, chi-square tests were used to examine associations between categorical variables, while the Mann-Whitney U test was applied to compare differences between two independent groups of continuous variables. A two-sided significance level of p ≤0.05 was set. Data analysis was performed using SPSS Statistics for Windows, v. 26 (IBM Corp., Armonk, NY, USA).

## Results

**Figure 5:**
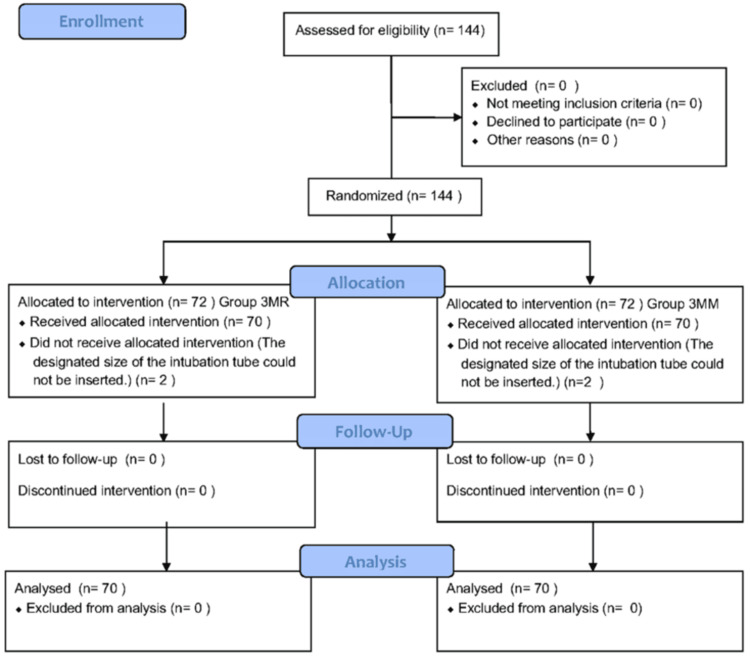
CONSORT flow diagram describing patient recruitment.

Between January and November 2024, a total of 144 patients were enrolled in this study. The CONSORT flow diagram shown in Figure 5 illustrates the process of participant recruitment and group allocation. All selected patients agreed to participate. The cohort was randomly assigned to either a 3MR or a 3MM group, based on the nasal pressure injury prevention method to be used during their surgery. During the trial, four of the patients withdrew, resulting in a final sample size of 140.

Evaluation of the condition of the nasal alar of each patient after extubation found a higher proportion of patients with no pressure marks or erythema in the 3MR group (45 patients) than the 3MM group (34 patients). The incidence of tube-induced erythema was lower in the 3MR group. Protective material-induced erythema was observed in six patients in the 3MR group and 11 in the 3MM group. However, no statistically significant difference was found between these comparisons (p = 0.66) (Table 1).

**Table 1:** Comparison of nasal alar after extubation between 3MR and 3MM groups (n). 1: Tube Imprint, 2: Erythema Due to Tube, 3: No Imprint or Erythema, 4: Erythema Due to Protective Material. 3MM: 3M Microfoam™ surgical tape, 3MR: 3M Reston™ self-adhesive foam pads

	1	2	3	4	P-value
3MR	0	19	45	6	0.66
3MM	3	22	34	11	

Next, we compared the PTN pressure between groups. The 3MR group had a median (interquartile range (IQR)) pressure of 51 (30.1-75.9) g, while the 3MM group had a median (IQR) pressure of 58.1 (36.1-116.8) g. Although this was slightly lower in the 3MR group, the difference was not statistically significant (p = 0.08) (Table 2).

**Table 2:** Comparison of pressure between intubation tube and nose (g). Median (interquartile range) 3MM: 3M Microfoam™ surgical tape, 3MR: 3M Reston™ self-adhesive foam pads

	Pressure				P-value
３MR	51 (30.1-75.9)				0.08
３MM	58.1 (36.1-116.8)				

The differences between nostril diameters and the outer diameter of the intubation tube were compared between groups. The median values were 1.8 (0.9-2.8) mm in the 3MR group and 1.8 (1.1-3.4) mm in the 3MM group, with no statistically significant difference between the two (p = 0.73) (Table 3).

**Table 3:** Comparison of nostril diameter and outer diameter of the intubation tube (mm). Median (interquartile range) OD: outer diameter, 3MM: 3M Microfoam™ surgical tape, 3MR: 3M Reston™ self-adhesive foam pads

	Diameter ー OD				P-value
3MR	1.8 (0.9-2.8)				0.73
3MM	1.8 (1.1-3.4)				

No significant differences were found in the median age, sex, height, or weight of the patients in the two groups. The duration of surgery and anesthesia also showed no significant differences, with the 3MR group averaging 120 (82.25-143.75) minutes and the 3MM group 107 (91-142) minutes (Table 4).

**Table 4:** Baseline demographic characteristics of 3MR and 3MM groups. Median (interquartile range) 3MM: 3M Microfoam™ surgical tape, 3MR: 3M Reston™ self-adhesive foam pads

	3MR			3MM			P-value
Sex (male/female）	44/26			32/38			0.06
								Age (year)	32 (25-42)			32 (27-42)			0.44
Height (cm)	166.5 (161-171)			164 (159-170)			0.85								
								Weight (kg)	60 (53-70.75)			59 (52-68)			0.46
Operation time (min)	73.5 (44.75-93.75)			66 (48-89)			0.77								
								Anesthesia time (min)	120 (82.25-143.75)			107 (91-142)			0.36

Throughout the surgical procedures, there were no incidents of 3MR or 3MM detachment. Additionally, no adverse events related to the use of either protective material were observed.

## Discussion

This study evaluated the comparative effectiveness of 3MR and 3MM in preventing nasal pressure injuries associated with NTI. We found that, while the 3MR group exhibited a lower incidence of tube-related erythema and pressure marks, as well as a tendency toward less pressure on the nasal alar, there were no statistically significant differences between the two groups.

A key finding was that the proportion of patients with no pressure marks or erythema was higher in the 3MR than the 3MM group. This suggests that 3MR may provide a more even distribution of pressure, reducing the localized stress on the nasal alar. The lower incidence of tube-related erythema in the 3MR group further supports this possibility. However, erythema caused by the protective material itself was observed in both groups, with a slightly higher incidence in the 3MM group.

The presence of protective material-induced erythema in both groups, despite the expectation that 3MM might cause more skin irritation, may be attributed to differences in material properties and pressure dynamics rather than direct skin contact. In this study, both 3MR and 3MM were directly attached to the endotracheal tube, not applied directly to the skin. Although 3MR has a softer and more adaptive structure, which might better conform to the nasal contour, both materials may still cause erythema due to factors such as movement during the procedure or individual skin sensitivity.

Both 3MR and 3MM were applied between the nasotracheal tube and the nasal alar rather than being directly attached to the skin. This placement was used to minimize the direct pressure from the tube on the nasal tissue. Despite this, differences in the properties of the materials may explain the observed trends. As a foam-based pad, 3MR has a softer, more adaptive structure that may conform better to the nasal contour, potentially reducing focal pressure points. In contrast, 3MM, while also cushioned, has a different degree of flexibility and pressure distribution, which could lead to slight differences in patient outcomes.

It should be noted that the tip of the 3MR or 3MM was intentionally placed inside the nasal cavity to protect the nasal alar during NTI. While mucosal contact was minimized to reduce potential irritation, no adverse effects related to this contact were observed during the study. This indicates that the method of protecting the nasal alar with the 3MR or 3MM was safe and well-tolerated by the patients.

Previous studies have emphasized the importance of MDRPI prevention. Kayser et al. demonstrated that MDRPIs represent a significant proportion of hospital-acquired pressure injuries, highlighting the need for effective countermeasures [15]. Furthermore, Brophy et al. systematically reviewed the incidence of MDRPI in acute hospital settings and concluded that improved pressure redistribution strategies are essential [16]. Our study suggests that 3MR may offer some advantage over 3MM by providing better pressure distribution and reducing direct skin irritation.

The PTN values in the 3MR group were slightly lower than those in the 3MM group, although the difference was not statistically significant. This trend is consistent with previous research that has shown the reduction of localized pressure to minimize tissue ischemia and subsequent pressure injury formation [7]. Pressure injuries are known to result from a combination of sustained pressure, shear forces, and microvascular compromise [8]. Therefore, pressure redistribution is vital to their prevention.

The differences between nostril diameters and the outer diameter of the intubation tube were also analyzed, and there was no significant difference between the two groups (p = 0.73). This suggests that, when selecting a tube for NTI, nostril diameter may not be a critical factor influencing the risk of nasal pressure injury. This could be because the use of protective materials (3MR and 3MM) mitigates the effects of tube pressure on the nasal alar, regardless of nostril size.

Although no statistically significant differences were observed, the trend toward less erythema and fewer pressure marks in the 3MR group suggests potential clinical benefits, particularly for patients with sensitive skin and those at high risk of MDRPIs. Given that pressure injuries often develop over time, using a material that reduces localized pressure may be beneficial in long-term applications. This would not have been evident in our results, so it warrants further exploration.

The absence of statistically significant differences in demographic factors, surgical duration, and anesthesia time indicates that these variables did not influence outcome differences between groups. This supports the validity of our comparisons between the 3MR and 3MM groups.

The present study had several limitations that should be considered. First, this was a single-center study, and this may limit the generalizability of our results. Second, while the study was adequately powered, subtle differences between the two materials may require a larger sample size to reach statistical significance. Third, we measured the FlexiForce ELF only once immediately after tracheal intubation to maintain consistency across patients. However, pressure changes during surgery due to factors such as neck movement or positional adjustments were not accounted for. Continuous measurement throughout the procedure may provide more comprehensive data on dynamic pressure changes. Fourth, we did not conduct either short or long-term follow-up, so it is unclear whether the protective effects of 3MR or 3MM extend beyond the immediate postoperative period. Previous studies on pressure injury prevention have emphasized the importance of longitudinal assessments to determine the durability of protective measures [10].

Future research should consider multicenter trials to improve the generalizability of the findings and use larger sample sizes to detect smaller but potentially clinically significant differences. Additionally, continuous intraoperative pressure monitoring could provide insights into pressure fluctuations caused by positional changes. Furthermore, studies incorporating long-term follow-up are needed to assess whether 3MR provides sustained benefits beyond the immediate postoperative period.

## Conclusions

Although we found no statistically significant differences between the 3MM and 3MR groups, the trends toward a lower incidence of erythema and fewer pressure marks in the 3MR group suggest that it may be beneficial for patients at higher risk of MDRPIs. Given the importance of even pressure distribution to the prevention of MDRPIs, further research into 3MR is warranted. This should include multicenter trials, larger sample sizes, and long-term follow-up to better assess the clinical impact of 3MR.
